# Supplementary material for: Application of CPI cutoff value based on parentage testing of duos and trios typed by four autosomal kits
Source: PLoS One. 2019 Nov 13;14(11):e0225174. doi: 10.1371/journal.pone.0225174 (PMC6853303; doi:10.1371/journal.pone.0225174)
Supplement: Attachment — Submitted filename: Response to reviewers.doc [file pone.0225174.s002.doc]

**Answers for comments raised in PONE-D-19-24597**

We are sorry for some flaws and incorrections appeared in this paper and appreciate the hard-work of your technical staff for revising. Here are the answers for the comments raised by the reviewers in the paper and we made changes in the paper accordingly.

**Reviewer #1:**

**1.** In the paragraph "STR loci mutations affect the conclusions of parentage testing with a low CPI value, and more autosomal STRs should be added to confirm the mutation" can you explain it's not clear written.
**Answer:** We explained it in discussion. “One or two STR loci mismatches may be due to mutational events. Sometimes, we should consider the mutational events as error events. Considering the possibility that autosomal STR loci mutations may occur, it is necessary to increase the number of the required STR loci and supplement the samples of the triplet. In this way, the identification errors could be greatly decreased.”

**2.** When you use abbreviations, for the first time in your manuscript, you have to give details about that.
**Answer:** we revised the abbreviations in the paper, including X-STRs and Y-STRs.

**3.** I recommend that a native speaker of English review the manuscript to improve word choice, sentence structure, and grammar.

**Answer:** The paper was revised by a native speaker of English (U.S.A).

**4.** The conclusion need to clear and specific, with 3 short conclusion.
**Answer:** We revised the conclusion according the suggestion of the reviewer.

**Reviewer #2:**

**1.** The language and structure of the article are so unclear that the merit can't be assessed. Most of the sentences are overly long, complicated, as well as ungrammatical in some instances. Throughout the article the language is neither clear, nor concise. Typographical and spelling errors have also been encountered.

**Answer:** The paper was revised by a native speaker of English (U.S.A).

**2.** The article gives relations to three cases that are not included in the study groups described in the methods. Therefore part of the article rather looks as a case report (in this case sample sizes are not large enough to produce robust results).

**Answer:**

(1) Three complex close relative kinship cases were analyzed to evaluate application of CPI Cutoff Value. At the same time, in order to explain the problem that high CPI value (CPI≥10000) will lead to false conclusions in a part of cases.

(2) In our present study, we only had 1442 real trio cases, 803 real duo cases and 3 complex close relative cases typed using Goldeneye 25A kit. We will study more cases in the future.

**3.** Regarding the tables of this manuscript, they are not all placed directly after the paragrapgh where they were first cited (see Table 2).
**Answer:** we revised it in the results and discussion.

**4.** The references formatting does not respect the style of the journal (does not list the first six authors followed by et.al.). Some of the title used as references are rather outdated (over 20 years old).

**Answer:** we revised the references formatting according to the style of the journal. One reference is over 20 years old (NO. 8), because DNA was extracted according to the method.
5. Laboratory protocols were not made fully availabe. Detailed protocols could enhance the reproductibility of the results.
**Answer:** We added the laboratory protocols in materials and methods (samples).

**6.** In part the discussions only repeat the results without interpreting them.

**Answer:** we amended the part of the discussion.
